# Sequence alterations in *RX* in patients with microphthalmia, anophthalmia, and coloboma

**Published:** 2009-01-21

**Authors:** Nikolas J.S. London, Patricia Kessler, Bryan Williams, Gayle J. Pauer, Stephanie A. Hagstrom, Elias I. Traboulsi

**Affiliations:** 1 Case Western Reserve University School of Medicine, Cleveland, OH; 2 Lerner Research Institute, Cleveland Clinic Foundation, Cleveland, OH; 3 Monash Institute of Medical Research, Monash University, Clayton, Victoria, Australia; 4 Department of Ophthalmic Research, Cole Eye Institute, Cleveland Clinic Foundation, Cleveland, OH; 5 Department of Ophthalmology, Cleveland Clinic Lerner College of Medicine of Case Western Reserve University, Cleveland, OH; 6 Department of Pediatric Ophthalmology and Strabismus, Cole Eye Institute, Cleveland Clinic Foundation, Cleveland, OH

## Abstract

**Purpose:**

Microphthalmia, anophthalmia, and coloboma are ocular malformations with a significant genetic component. *Rx* is a homeobox gene expressed early in the developing retina and is important in retinal cell fate specification as well as stem cell proliferation. We screened a group of 24 patients with microphthalmia, coloboma, and/or anophthalmia for *RX* mutations.

**Methods:**

We used standard PCR and automated sequencing techniques to amplify and sequence each of the three *RX* exons. Patients’ charts were reviewed for clinical information. The pathologic impact of the identified sequence variant was analyzed by computational methods using PolyPhen and PMut algorithms.

**Results:**

In addition to the polymorphisms we identified a single patient with coloboma having a heterozygous nucleotide change (g.197G>C) in the first exon that results in a missense mutation of arginine to threonine at amino acid position 66 (R66T). In silico analysis predicted R66T to be a deleterious mutation.

**Conclusions:**

Sequence variations in *RX* are uncommon in patients with congenital ocular malformations, but may play a role in disease pathogenesis. We observed a missense mutation in *RX* in a patient with a small, typical chorioretinal coloboma, and postulate that the mutation is responsible for the patient’s phenotype.

## Introduction

Eye development is an intricate process that occurs early in embryogenesis and is governed by a highly organized sequence of genetic events. Perturbation of these events may result in a wide range of congenital eye malformations. Microphthalmia, anophthalmia, and coloboma are examples of such malformations, and are thought to have significant genetic components [1-6].

*Rx* is a paired-domain homeobox gene that is essential for vertebrate eye development. It is strongly expressed in the retina and anterior neural fold during early embryogenesis. Its product is a transcription factor that directs initial retinal cell specification and subsequent proliferation, and is expressed weakly in the adult retina, restricted to the zone of proliferating cells [7]. *Rx* gene structure and protein homeodomains are functionally conserved among species [7-12], and *Rx* appears to be essential for proper vertebrate eye development [9,10,13-16]. Retinal development involves a complex orchestration of gene activation and expression. Nrl, Rx, and Pax6 are 3 of many important transcription factors. These also include *CHX10, CRX, ET, Six3, Optx2, Tlx*, and *Lhx2*. These genes form a genetic network that is largely conserved from flies to humans. Rx is one of the earliest of these to be expressed, and is essential for normal eye development.

*Rx* increases the transcription of other eye-specific genes, including *Pax6*. However, the direct interactions between *Rx* and *Pax6* have not been elucidated. Neural retina leucine zipper (Nrl) is a basic leucine zipper protein of the Maf subfamily that is preferentially expressed in rod photoreceptors. It acts synergistically with the homeodomain protein CRX to regulate rhodopsin transcription. Direct interactions have been shown between Nrl and CRX. Targeted homozygous deletion of the *Rx* gene in the mouse results in anophthalmia [7]. *Rx* mutations have been identified in several animal models of anophthalmia, including mouse [14], medaka (the Japanese killfish) [11], zebrafish [17], and *Xenopus* [18,19]. Rx protein homology is well conserved, and is identical in *Xenopus*, *Drosophila,* and zebrafish.

Voronina et al. [2] identified and characterized compound heterozygous *RX* mutations in a patient with autosomal recessive microphthalmia of one eye and sclerocornea of the other. The patient had a truncated allele (Q147X), which affected nuclear localization, and a missense mutation (R192Q), which affected DNA-binding. Both alleles were within the homeodomain of the RX protein and were likely sufficient to account for the patient’s phenotype. The researchers also reported two polymorphisms (E44/D44 and Q294Q) in the first and third exons of *RX*. More recently, Lequeux and coworkers [20] confirmed the involvement of *RX* in human anophthalmia by reporting a patient with bilateral anophthalmia and compound heterozygous mutations. Our study sought to build upon these findings and to further examine the role of *RX* in congenital ocular malformations. We screened a group of 24 patients with colobomatous microphthalmia for *RX* mutations, and correlated our findings with the clinical phenotype of the patients.

## Methods

### Patients

This study was approved by the Internal Review Board of the Cleveland Clinic Foundation. All blood samples were obtained after informed consent was secured. A total of 24 index patients were studied: 2 with a positive family history while the remaining 22 had no known immediate family history of ocular malformations. These are all patients microphthalmia, anophthalmia, and colobomas who have consented to participate in a study of the genetics of eye diseases at the Cole Eye institute. All patients received diagnoses through ophthalmologic examination. Patients’ charts were reviewed for clinical information (Appendix 1). A total of 222 unrelated individuals without symptoms or a family history of retinal disease were used as normal control subjects. The controls were recruited from patients and their families who visit the Cole Eye Institute for routine eye care. All have signed informed consent to participate in this study.

### Mutation detection

Leukocyte nuclei were prepared from blood samples followed by DNA purification using standard protocols. Five ml of venous blood was drawn from each individual. Leukocyte nuclei were purified and DNA was extracted using Gentra Puregene® Blood Kit before PCR analysis. PCR products corresponding to the complete known *RX* coding sequence (NM_013833) were amplified from genomic DNA and analyzed by direct sequencing using an automated sequencer. The primers described in Table 1. PCR reactions were tailored to each primer pair to yield optimal amplification. Cycling conditions were as follows: denaturation at 95 °C for 5 min, 40 cycles of 94 °C for 60 s, 59 °C for 30 s, and 74 °C for 45 s, and a final extension at 74 °C for 5 min.

**Table 1:** Listing of PCR primers used to amplify *RX* in this study.

**Exon**	**Sense primer**	**Antisense primer**
1	GGGCGCCCGAACGGCCCTC	GCCTCTCCTCTCCGTCTCC
2	GGAGTGCATCTGACCCTCC	TGGCTGCAATTTGGGCCTCG
3	GAGCTGAACCGGCTCAGG	GGATCCCAAGACGTTCCCC

PCR products were gel-purified. They were then directly sequenced, using the SequiTherm Excel II DNA sequencing kit (Epicenter Technologies, Madison, WI), on an automated sequencer (3130XL; Applied Biosystems, Foster City, CA).

### Computational assessment of R66T

Two sequence homology based programs were used to predict the functional impact of R66T: PolyPhen (polymorphism phenotyping) and PMut. PolyPhen structurally analyzes an amino acid polymorphism and predicts whether that amino acid change is likely to be deleterious to protein function [21]. The prediction is based on the position specific independent counts (PSIC) score derived from multiple sequence alignments of observations. PolyPhen scores of >2.0 indicate the polymorphism is probably damaging to protein function; scores of 1.5–2.0 are possibly damaging; and scores of <1.5 are likely benign. PMut allows the accurate pathological prediction of single amino acid mutations based on the use of neural networks [22]. Following the input of a reference sequence and the amino acid substitution of interest, the algorithm provides an answer and a reliability index. An output value >0.5 is predicted to be a pathological mutation and a value <0.5 is neutral. The reliability is considered good with a score of 6 and greater and is highly reliable at the maximum score of 9.

## Results

Malformations represented by our patient population included 10 diagnosed with isolated coloboma, 6 with colobomatous microphthalmia, 5 with isolated microphthalmia, 1 with microphthalmia and anophthalmia, 1 with anophthalmia, and 1 with nanophthalmos (Appendix 1).

We identified 2 polymorphisms (E44/D44 and Q294Q) that have previously been described by Voronina et al. [2] The first (E44/D44) involves either glutamic acid or aspartic acid being found at amino acid 44 of exon 1. The second (Q294Q) is a silent mutation with either A or G in the third codon position of amino acid 294 in exon 3. Of the 24 patients, 7 carried the E44/D44 polymorphism, 6 the Q294Q polymorphism, and 4 patients carried both (Appendix 1). We compared the patient’s phenotypes with the observed nucleotide variations. Diagnoses among the 7 patients carrying the E44/D44 polymorphism consisted of isolated coloboma (3), microphthalmia (2), colobomatous microphthalmia (1), and combined anophthalmia–microphthalmia (1). Diagnoses among the 6 patients carrying the Q294Q polymorphism consisted of coloboma (5) and microphthalmia (1).

**Figure 1:**
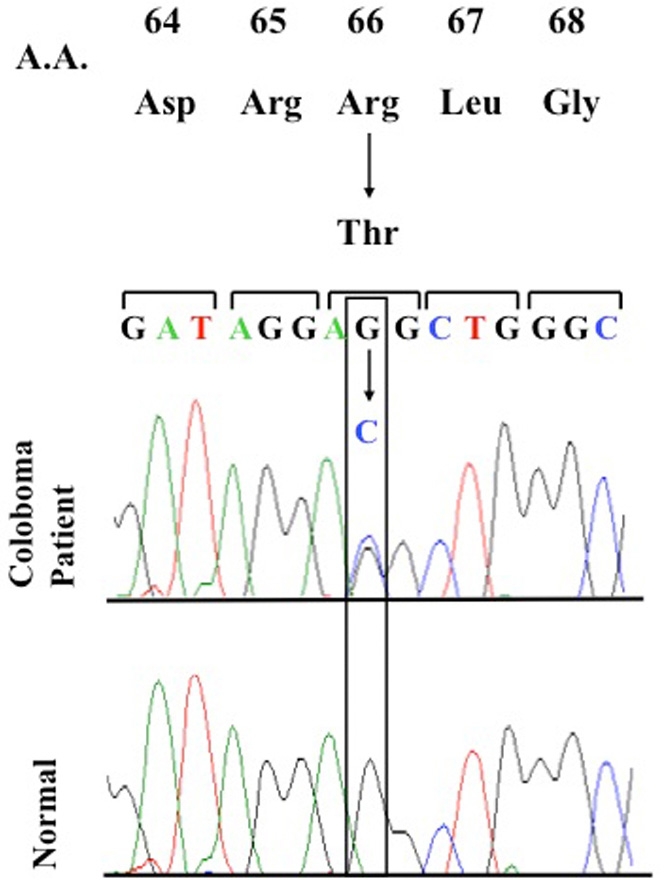
DNA sequence of the *RX* heterozygous R66T mutation. DNA sequence of the *RX* heterozygous mutation R66T in exon 1 in a coloboma patient and the corresponding DNA sequence in a control individual. Abbreviations: A.A. is Amino acid, Asp is aspartic acid, Arg is arginine, Leu is leucine, Gly is glycine.

**Figure 2:**
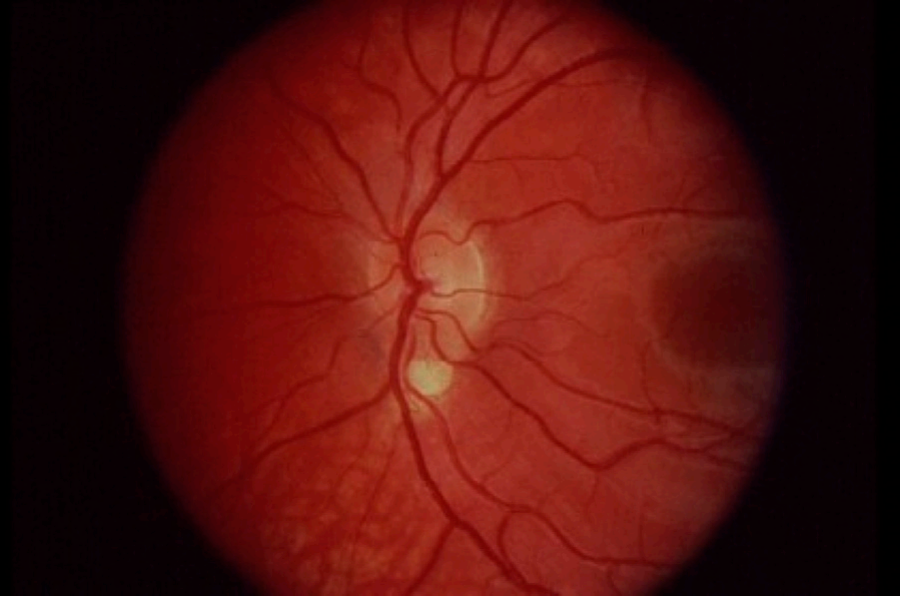
Fundus photographs of the patient with *RX* mutation, R66T. Right fundus with small typical coloboma at the inferior edge of the nerve that is vertically elongated. Note the pattern of exit of the inferior arcade vessels, indicating the presence of the coloboma.

In addition, we identified a single patient with a heterozygous nucleotide change, G>C at nucleotide position 197 in the first exon, that results in a missense mutation of arginine to threonine at amino acid position 66 (R66T; Figure 1). The patient also carried both polymorphisms. The patient’s ocular phenotype was a small, typical retinal coloboma of the right eye (Figure 2). In silico analysis using PolyPhenpredicted R66T to be possibly damaging to the protein. A large difference (1.602) was noted in PSIC scores between the allelic variants arginine versus threonine. This difference indicates that the observed substitution is rarely or never observed in the RX protein family and is predictive of a structurally damaging mutation that alters the function of the protein. PMutanalysis also predicts R66T to be pathological with high reliability.

We analyzed the DNA of 222 unaffected individuals and did not find the g.197G>C mutation in these patients. We did observe the polymorphisms reported by Voronina et al. [2] (E44/D44 and Q294Q) in both patients and controls. We did not observe either of previously described mutations (Q147X or R192Q) in any of the patients.

## Discussion

It is the complexity of eye development that underlies the diversity of structural eye disease, with numerous opportunities for disruption [23]. Environmental factors have been suggested to play a role in the malformations, including exposure to infections as well as toxins during pregnancy [24-28]. More intensely scrutinized, however, are the genetic contributions. Several genes known to be important in ocular development have been implicated, including *PAX6, RX, SOX2, OTX2, CHX10, PAX2, SHH,* and *SIX6*. We found a missense mutation in *RX* in a patient with a small, typical retinal coloboma. *RX* mutations have been associated with primarily severe ocular malformations such as anophthalmia [2,20]. Our findings suggest a possible role for *RX* in later developmental stages that have not previously been described, particularly latter-stage closure of the optic fissure. Unfortunately family members of our patient were not examined; therefore we cannot comment on the inheritance pattern of this mutation. While the 2 previous reports suggest a recessive mode for severe ocular malformations such as anophthalmia, we believe that there may be a dosage effect of this gene. When 2 pathogenic mutations are present [2,20], anophthalmia or severe microphthalmia is produced. In our case, this single mutation results in a reduced dosage of the protein and leads to a minor malformation, a small coloboma at the optic nerve head.

Several genetic mutations have been implicated in microphthalmia and anophthalmia. Glaser et al. [1] described homozygous loss of function mutations in *PAX6* in a patient with anophthalmia and central nervous system malformations. Mutations in *SOX2* cause predominantly anophthalmia [6,29,30]. Mutations in *OTX2* have been associated with various severe eye malformations, including anophthalmia and microphthalmia [31]. *PAX2* malformations have been associated with optic nerve coloboma [4,5,32], *SIX6* mutations with bilateral anophthalmia and pituitary abnormalities [33] as well as microphthalmia [34], *CHX10* mutations with microphthalmia [3], and *SHH* mutations with human microphthalmia and coloboma [35].

*Rx* gene structure and protein homeodomains are well conserved among species [7-12], and animal studies have established *Rx* as a high-order gene in ocular development. Mathers et al. [7] showed in knockout studies in mice that elimination of *Rx* prevented eye formation. In a zebrafish model of anophthalmia (*chokh* mutation), Loosli et al. [11] showed that the mutation resulted from a nonsense mutation in the homeodomain of *Rx3*, leading to a severely truncated protein. Tucker et al. [14] established that the mouse eyeless mutation is secondary to a disrupted translation initiation site leading to reduced levels of Rx protein. Andreazzoli et al. [18] showed that the *Xenopus* model of anophthalmia is due to elimination of Rx function.

In human subjects, sequence variations in *RX* are uncommon in patients with major congenital ocular malformations, but may play a role in the pathogenesis of these malformations. Voronina et al. [2] described a patient with anophthalmia OD and sclerocornea OS who was a compound heterozygote for *RX* mutations. The patient had a truncated allele (Q147X), which affected nuclear localization, and a missense mutation (R192Q), which affected DNA binding. Both alleles were within the homeodomain of the RX protein and were likely sufficient to account for the patient’s phenotype. Lequeux et al. [20] report a patient with bilateral anophthalmia and two mutations in exon 3 (c.664delT and c.909C>G) that lead to premature truncation of the protein,

We describe a heterozygous missense mutation in the first exon of *RX* in a patient with a small, typical optic nerve coloboma of the right eye. We postulate that the mutation is responsible for the patient’s phenotype. Given the importance of *Rx* in eye development, it is possible that even a small perturbation would result in an ocular defect. While the majority of research delineates the role of *Rx* in the initial stages of ocular development, and the majority of functional studies have been knockout experiments that resulted in severe malformations, there is also evidence that *Rx* plays a role in the later stages of retinal development. Our finding strengthens this evidence and implies that *Rx* plays a role in closure of the optic fissure in addition to its other established roles. It is possible that the location of the sequence variation outside the homeobox or the paired box may explain the mild nature of the colobomatous defect. A sequence variation/mutation within the homeobox or the paired box may have caused a more severe phenotype.

The g.197G>C nucleotide change results in an alteration in the primary amino acid sequence from a large, positively-charged amino acid (arginine) to a small, neutral amino acid (threonine).While we have yet to characterize the functional consequences of this change, it is conceivable that the secondary and tertiary structures are sufficiently affected to alter the function of the protein. To predict the potential effect of the R66T missense change, we obtained estimates of the impact of this mutation through the use of two sequence homology-based programs. Both the PolyPhenand PMutalgorithms predict this amino acid change to be structurally damaging to the RAX protein. However, it is also possible that the mutation we discovered in this patient is not related to the phenotype, but is rather a coincidental finding.

## Appendix 1. Clinical details of all cases.

Table summarizing clinical details and *RX* sequence variations in 24 patients with ocular malformations of the microphthalmia/coloboma/anophthalmia spectrum. Abbreviations: male (M), female (F), autosomal dominant (AD), right eye, (OD), left eye (OS), both eyes (OU). To access the data, click or select the words “Appendix 1.” This will initiate the download of a compressed (pdf) archive that contains the file.
